# First person – Karla G. Espinosa and Salma Geissah

**DOI:** 10.1242/dmm.049578

**Published:** 2022-05-09

## Abstract

First Person is a series of interviews with the first authors of a selection of papers published in Disease Models & Mechanisms, helping early-career researchers promote themselves alongside their papers. Karla G. Espinosa and Salma Geissah are co-first authors on ‘ Characterization of a novel zebrafish model of *SPEG*-related centronuclear myopathy’, published in DMM. Karla completed the research described in this article while an MSc student in the lab of Dr James J. Dowling at the Hospital for Sick Children and University of Toronto, Toronto, ON, Canada, investigating the molecular mechanisms behind cardiac and skeletal muscle contraction. Salma is an MSc student in the lab of Dr James J. Dowling at the Hospital for Sick Children and University of Toronto, Toronto, ON, Canada, investigating the molecular biology of muscle disease, how muscles function and the dynamic processes involved.

**Figure:**
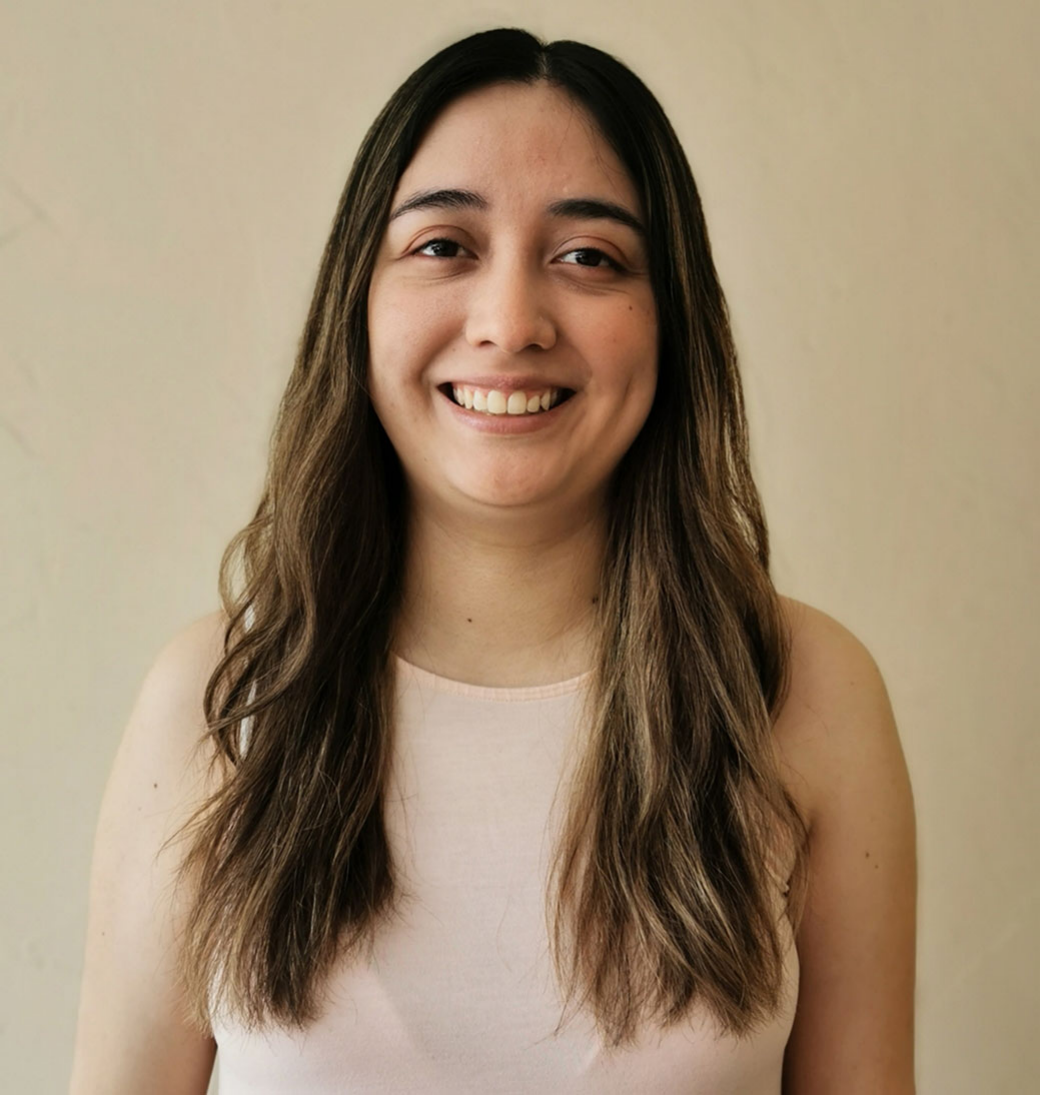
Karla G. Espinosa

**How would you explain the main findings of your paper to non-scientific family and friends?**

KGE: We developed a novel animal model to study how the absence of a protein called SPEG causes a disease known as centronuclear myopathy (CNM). This disease can cause children to have muscle weakness and cardiac problems, with the most severe cases resulting in early mortality. We decided to generate this new model in zebrafish because early developmental studies are not currently possible in mice due to early lethality. We found that our novel zebrafish *spega/b* mutant model shows defects similar to those previously reported in CNM, including abnormal muscle structure, decreased movement and shorter lifespan, thus demonstrating that our new zebrafish mutants faithfully model the CNM disease. Next, we compared if the absence of SPEG was similar to the absence of other proteins such as MTM1 and DNM2 since these two are also associated with some cases of CNM. One similarity we found in these three CNM models was the accumulation of the protein desmin in the myofibres. On the other hand, only the disruption of SPEG or MTM1 resulted in higher levels of the protein DNM2. Overall, we found that the absence of SPEG results in muscles not being able to organize, build and contract as they should, which causes motor problems and decreased survival. Finally, thanks to our novel zebrafish model, we can better understand the disease mechanisms behind CNM and work on identifying potential therapies for these patients.

SG: CNM is a devastating childhood muscle disorder with no currently approved treatments, and this is partly attributable to an incomplete understanding of the disease. There are various CNM subtypes caused by mutations in five different genes, including the striated preferentially expressed kinase (*SPEG*) gene. To better understand CNM, we generated a CNM zebrafish model by using CRISPR/Cas9 technology to model patient mutations in the *SPEG* gene. Our SPEG-CNM zebrafish model replicates all the main symptoms found in CNM patients. We found that these fish have a decreased lifespan, reduced muscle function and structural abnormalities in muscle structures. We also compared our new SPEG-CNM model to two other CNM zebrafish models. These comparisons allowed us to find molecular similarities between the CNM subtypes, which could lead to a better understanding of disease development and hopefully therapies.

**Figure:**
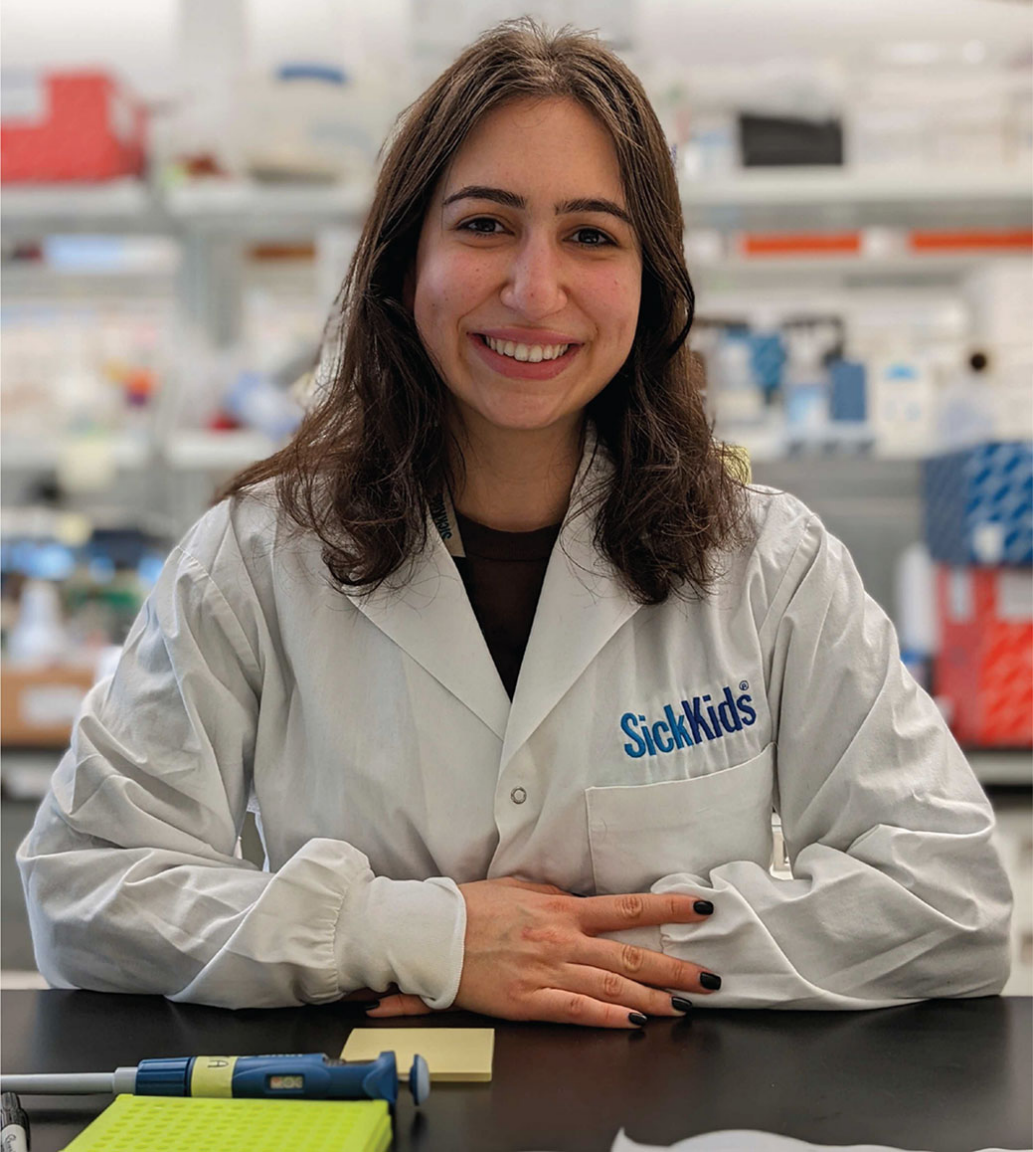
Salma Geissah

**Figure:**
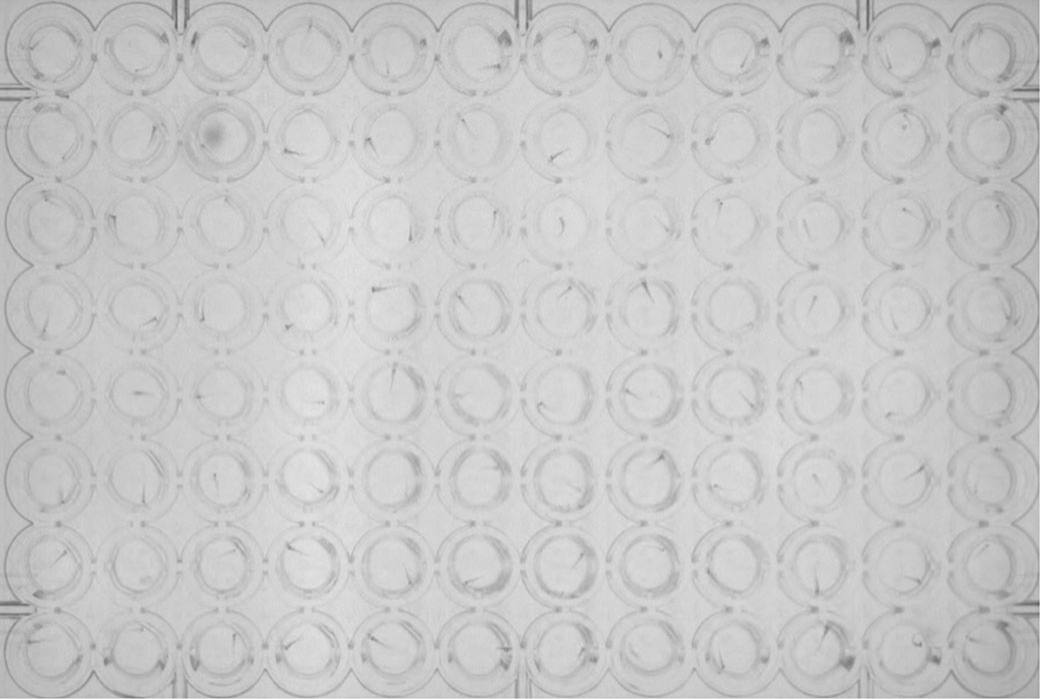
Sample layout of how zebrafish embryos (either at 3 or 5 days post-fertilization) are individually placed in 96-well plates to perform swimming assays using Zebrabox (Viewpoint, France).

**What are the potential implications of these results for your field of research?**

KGE: Currently, there is no treatment for patients with CNM, so this novel animal model provides us with the opportunity to perform large-scale drug experiments to identify potential drug treatments for these patients. This new model also allows us to increase our understanding of the molecular mechanisms behind muscle development and function, including when and where some excitation–contraction coupling interactions occur, and we can do so in shorter periods of time compared to when using mouse models. Additionally, the similarities we found between different CNM models (SPEG, MTM1 and DNM2) suggest that they have common downstream proteins that could be modified either through genetics or with chemicals to rescue (or ameliorate) the mutant phenotypes. Therefore, it remains possible that once we find a treatment for one of them, it could also work for other (or all) CNM patients.

SG: CNM is a developmental disease, so it's critical to investigate early skeletal muscle development to better understand the disease pathology. We generated the first SPEG-CNM zebrafish model, which survives up to 12 days post-fertilization and faithfully recapitulates phenotypes observed in patients and mouse models, allowing us to focus on studying SPEG's role in early skeletal muscle development and how its loss can lead to CNM. We also found that SPEG-CNM has comparable disease markers to other CNM subtypes. This exciting model gives us the opportunity to further investigate these disease makers throughout early development and will enable large-scale chemical screens to hopefully identify putative drug targets for this devastating disease. I also hope that the strong conservation of phenotypes across patients, mouse models and now zebrafish will provide further evidence on how great a model zebrafish can be for human diseases!

**What are the main advantages and drawbacks of the model system you have used as it relates to the disease you are investigating?**

KGE: CNM usually presents at birth and some of these patients have cardiac and skeletal muscle defects. Our model allows studying both tissues at the same time using constitutive SPEG knockouts (where SPEG is always disrupted in all tissues) because zebrafish does not rely on a fully functional heart during the first 7 days post-fertilization. This means we can do live imaging, *in vivo* studies and whole-embryo experiments during early embryogenesis even if our mutants present cardiac defects. We can also evaluate the progress of CNM muscle defects during development by quantitatively assessing changes in muscle structure and function. Another main advantage is that we can now perform large-scale drug screenings to identify chemicals that rescue CNM defects, which is key due to the lack of treatments for these patients. Additionally, it is faster to evaluate interactions between CNM genes and we can later use this information to test if knocking out or overexpressing additional genes ameliorates the mutant phenotypes. Overall, this new zebrafish model helps us better understand the pathological mechanisms behind CNM, and we can perform more experiments in shorter periods of time. As for disadvantages, our model has not presented cardiac defects at the stages examined, so it is still unclear if it could be used to evaluate any possible relationships between the cardiac and skeletal muscle defects.

SG: Zebrafish are extremely advantageous to us because they can survive several days post-fertilization with impaired muscle function, making it possible to study early muscle development. Their skeletal muscle structure is also very similar to that of humans. Moreover, because of the transparent nature of zebrafish embryos, we are also capable of tagging proteins of interest with fluorescent markers, allowing us to study protein dynamics, localization and interaction *in vivo* and at different stages throughout development. Another critical advantage of our model system is that the use of zebrafish enables us to conduct large-scale drug screens, making it possible to screen for hundreds of drugs in an *in-vivo* model for CNM.

Although, one drawback to using zebrafish is the limitation we have with finding validated reagents. Specifically, it's a challenge to find antibodies that work for different molecular techniques, making the optimization process a little bit more troublesome.

“Seeing zebrafish replicating the defects reported in *SPEG*-related CNM was amazing because it meant this novel zebrafish model can be used to study CNM and to find potential drug therapies.”

**What has surprised you the most while conducting your research?**

KGE: I remember two moments in which I was very surprised. The first one was when I learned that zebrafish can survive up to 7 days post-fertilization without a functional heart. The second one was when I was characterizing the phenotypes in the single knockouts (KOs). When working with zebrafish genes that have paralogs (gene copies derived from a duplication event), it is not always possible to see mutant phenotypes in the single KOs, especially when the paralogs are expressed in similar tissues. This means that sometimes you have to wait until all paralogs are disrupted before seeing any defects in the mutants. Therefore, I was very excited when the *spegb* single KOs showed strong mutant phenotypes such as earlier mortality and swimming defects that led to their paralysis. Seeing zebrafish replicating the defects reported in *SPEG*-related CNM was amazing because it meant this novel zebrafish model can be used to study CNM and to find potential drug therapies.

SG: Something that never fails to amaze me is how scientists have developed all these techniques to be able to visualize and quantify such tiny molecules in an extremely specific and accurate manner, allowing us to dissect the roles of singular proteins in an entire organism! Specifically, looking at protein localization in zebrafish muscle under the confocal microscope is always fascinating to look at. It's very interesting to observe the evolutionary conservation of these molecular structures.

**Describe what you think is the most significant challenge impacting your research at this time and how will this be addressed over the next 10 years?**

KGE: The most significant challenge at this point is to understand the molecular mechanisms behind CNM to be able to find a treatment for these patients. While some proteins associated with this disease have been identified, we still don't fully understand the role they play in muscle development and function. We also don't know all their interacting partners and the order in which some processes occur. It is unclear whether we have to correct all those disrupted interactions to rescue the mutant phenotypes or if correcting a single interaction is enough to prevent all CNM. Over the next 10 years, this will be addressed using proteomic, genomic and pharmacological approaches. Proteomics will be used to identify all interacting partners of CNM proteins, including SPEG. Genomic approaches will help evaluate which interactions are critical to rescue the CNM phenotypes. Ultimately, pharmacological approaches will let us explore additional pathways that could play a role in the progress or delay of this disease, while also looking for possible drug treatments for these patients.

SG: I think a major challenge (but also a plus) for our research now is that very little is known about the kinase functions of our protein of interest. To address this, we aim to conduct experiments that will generate relatively large datasets that we will have to comb through to identify what is biologically relevant. One of the ways we will be validating our top hits from these data sets is by generating mutant zebrafish lines using CRISPR/Cas9 technology to functionally assess the effects of these targets. This process could be very costly and time consuming, especially if we want to generate multiple lines. Over the next 10 years, I think that mutagenesis will only get easier and faster to do, hopefully allowing us to functionally validate several targets and further understand skeletal muscle development and disease.

**What changes do you think could improve the professional lives of early-career scientists?**

KGE: One of the challenges that early-career scientists overcome is measuring their success or their potential based on the number of publications they have or based on the number of grants or fellowships they win. These criteria could leave great early-career scientists behind when they start to build their professional lives, including those starting new projects from zero, those whose progress is limited by the current resources of their laboratories/countries, and those for whom family/personal reasons must temporarily pause their presence in the laboratories (e.g. parental leave, mental health). Some ways to help them overcome these obstacles could include promoting the inclusion of early-career scientists in publications, assigning more money for research grants and increasing the number of fellowships for early-career scientists. Perhaps slightly less money per fellowship but helping more young scientists would be more helpful. The constant pressure to publish and get grants could lead to scientists spending less time doing research and could eventually push them away from science instead of making them stay.

SG: I think I am very fortunate to be an early-career scientist in the Dowling lab, SickKids and University of Toronto, as we are always being provided with various opportunities. Our PI is always encouraging us to submit abstracts to conferences, participate in seminars and take part in the grant writing process, and the department has an amazing system that helps keep your project on track with regular faculty committee meetings. Although something that I think could be improved is having more exposure to different careers in science and having some sort of career mentorship throughout graduate programs.

**What's next for you?**

KGE: I'm highly interested in understanding the molecular mechanisms behind human diseases, as well as in developing new and better treatments for these. Therefore, I would love to do a PhD to keep exploring the causes of cardiac and skeletal muscle defects, and to evaluate how we could prevent them or treat them. My goal is to keep doing research in the coming years and to include mentorship and teaching in my plans. Likely, I'll stay in academia to later become a PI and faculty professor. Additionally, I would like to collaborate at some point with the industry to help pharmaceutical companies in the development and testing of drug treatments for patients with these conditions.

SG: Right now, I'm very excited to be transitioning from an MSc candidate to a PhD candidate in the Dowling lab. I will be continuing to work on exploring SPEG's role in skeletal muscles throughout development and in CNM over the next couple of years. I hope we can identify some of SPEG's kinase substrates that we can target via drugs or genetic therapies to help treat CNM.

“[…] collaborations are very important not only to optimize research resources, but also to improve our abilities as scientists and to open doors for future projects/jobs.”

**How important do you think collaborations are in science?**

KGE: I'm very glad I had the opportunity to collaborate with experts of cardiac development (Dr Ian C. Scott) and calcium transients (Dr Robert T. Dirksen and Dr Linda Groom) because this allowed me to improve my critical thinking and scientific knowledge. We were also able to perform more experiments and to analyse the results as best as possible. I'm also thankful I met Professor Scott when I was an undergraduate student because that connection helped me to later join the Dowling group where I worked on this research. As a young scientist, I can say that collaborations are very important not only to optimize research resources, but also to improve our abilities as scientists and to open doors for future projects/jobs.

SG: I think collaborations are what makes research so powerful. I love being in an environment where we are constantly working as a team with people from all over the world and with a vast diversity in expertise. I am constantly being exposed to different ways of thinking about problems, techniques and models. I'd also like to take this opportunity to thank Dr Dirksen, Dr Groom and Dr Scott for not only their help with experiments but also the constant feedback and invaluable input they provided and continue to provide.
